# Periconceptional ultra-processed food consumption in women and men, fertility, and early embryonic development

**DOI:** 10.1093/humrep/deag023

**Published:** 2026-03-24

**Authors:** Celine H X Lin, Romy Gaillard, Annemarie G M G J Mulders, Vincent W V Jaddoe, Mireille C Schipper

**Affiliations:** The Generation R Study Group, Erasmus MC, University Medical Center, Rotterdam, the Netherlands; Department of Pediatrics, Sophia’s Children’s Hospital, Erasmus MC, University Medical Center, Rotterdam, the Netherlands; The Generation R Study Group, Erasmus MC, University Medical Center, Rotterdam, the Netherlands; Department of Pediatrics, Sophia’s Children’s Hospital, Erasmus MC, University Medical Center, Rotterdam, the Netherlands; Department of Obstetrics and Gynecology, Erasmus MC, University Medical Center, Rotterdam, the Netherlands; The Generation R Study Group, Erasmus MC, University Medical Center, Rotterdam, the Netherlands; Department of Pediatrics, Sophia’s Children’s Hospital, Erasmus MC, University Medical Center, Rotterdam, the Netherlands; The Generation R Study Group, Erasmus MC, University Medical Center, Rotterdam, the Netherlands; Department of Pediatrics, Sophia’s Children’s Hospital, Erasmus MC, University Medical Center, Rotterdam, the Netherlands

**Keywords:** periconception, ultra-processed foods, diet, fertility, CRL, yolk sac

## Abstract

**STUDY QUESTION:**

Is periconceptional ultra-processed food (UPF) consumption in women and men associated with fertility, embryonic growth, and yolk sac development?

**SUMMARY ANSWER:**

Higher maternal UPF consumption was associated with smaller embryonic growth and yolk sac volume, and higher paternal UPF consumption was associated with reduced fertility.

**WHAT IS KNOWN ALREADY:**

The periconceptional period is critical for the probability of conception, pregnancy outcomes, and long-term offspring health, and maternal dietary patterns during this period may influence fertility as well as early embryonic development. No studies have examined the combined influence of maternal and paternal UPF consumption on fertility outcomes and early development.

**STUDY DESIGN, SIZE, DURATION:**

This study is embedded in a population-based prospective cohort study from preconception onwards, with follow-up into childhood. The present study included 831 women and 651 male partners.

**PARTICIPANTS/MATERIALS, SETTING, METHODS:**

We assessed periconceptional dietary intake via a food frequency questionnaire at median 12^+3 ^weeks of gestation (95% range 10^+6^, 18^+3^). Foods were categorized using NOVA classification, and UPF intake was expressed as a percentage of total food intake (grams per day). Information on time to pregnancy was obtained through questionnaires, with fecundability defined as the probability of conceiving within 1 month and subfertility as time to pregnancy ≥12 months or use of assisted reproductive technology. Crown-rump length (CRL) and yolk sac volume were measured by transvaginal ultrasound examinations at 7, 9, and 11 weeks of gestation.

**MAIN RESULTS AND THE ROLE OF CHANCE:**

Median UPF intake was 22.0% of total food intake for women and 25.1% for men. UPF intake was not associated with fertility outcomes in women. In fully adjusted continuous models, higher maternal UPF intake was associated with smaller CRL at 7 weeks gestation [difference: −0.13 standard deviation score (SDS), 95% CI: −0.25, −0.01, per SDS increase in UPF intake]. Higher maternal UPF intake was also associated with smaller yolk sac volume at 7 weeks gestation in fully adjusted continuous models [difference: −0.14 SDS, 95% CI: −0.26, −0.02, per SDS increase in UPF intake]. These associations attenuated at 9 and 11 weeks gestation. In men, higher UPF intake was associated with decreased fecundability and increased subfertility risk in the continuous models (fecundability ratio: 0.90, 95% CI: 0.83, 0.99, OR: 1.36, 95% CI: 1.11, 1.67, per SDS increase in UPF intake), but not with first trimester development, after adjusting for socio-demographic, lifestyle factors, and female partner’s UPF consumption.

**LIMITATIONS, REASONS FOR CAUTION:**

The inclusion of a relatively healthy population may limit the generalizability of our findings to broader or higher-risk groups.

**WIDER IMPLICATIONS OF THE FINDINGS:**

These sex-specific findings underscore the importance of considering UPF intake in preconception and early pregnancy diets to optimize reproductive outcomes, embryonic development, and long-term offspring health.

**STUDY FUNDING/COMPETING INTEREST(S):**

The Generation R Study is financially supported by the Erasmus Medical Center, Rotterdam, the Erasmus University Rotterdam, and the Netherlands Organization for Health Research and Development. R.G. received funding from the Netherlands Organization for Health Research and Development (NWO, ZonMw VIDI 09150172110034, and NWO, ZonMW, grant number 05430052110007), from the Dutch Diabetes Foundation (grant no. 2024.28.001), and from the European Union (ERC, OBESE-EMBRYO, ERC-2024-STG-101161004). V.W.V.J received a grant from the Netherlands Organization for Health Research and Development (NWO, ZonMw 05430052110007) and a European Research Council Consolidator Grant (ERC-2014-CoG-648916). Views and opinions expressed are however those of the author(s) only and do not necessarily reflect those of the European Union or the European Research Council. Neither the European Union nor the granting authority can be held responsible for them. This project has received funding from the European Union’s Horizon 2020 research and innovation program under the ERA-NET Cofund action (No. 727565), European Joint Programming Initiative “A Healthy Diet for a Healthy Life” (JPI HDHL), EndObesity, ZonMW Netherlands (No. 529051026). The authors declare that they have no conflict of interest.

**TRIAL REGISTRATION NUMBER:**

N/A.

## Introduction

The first 1000 days of life, spanning from conception through the second year, represent a critical window during which biological processes lay the foundation for growth, development, and long-term health (Indrio *et al.*, 2023). The preconception period and early pregnancy are particularly sensitive to adverse environmental influences, which may adversely impact reproductive success and embryonic development, with consequences that may last a lifetime (Hoffman *et al.*, 2017). Suboptimal reproductive health is a growing global concern that imposes significant physical, emotional, and financial burden on affected individuals (Taylor, 2003; Sun *et al.*, 2019; Cox *et al.*, 2022). Early development is characterized by rapid development and growth of the embryo and the yolk sac. Impaired first-trimester embryonic growth is associated with an increased risk of adverse birth outcomes, including preterm birth, low birth weight, and an unfavorable cardiovascular profile in childhood (Mook-Kanamori *et al.*, 2010; Jaddoe *et al.*, 2014). The yolk sac plays a critical role in nutrient delivery to the embryo before placental function is fully established, and impaired yolk sac development is associated with increased risk of miscarriage and preterm birth (Donovan, 2025). To mitigate these risks, it is crucial to identify modifiable factors that influence reproductive success and early development in women and men (Wu *et al.*, 2004; Barker, 2007; Rossi *et al.*, 2014).

In recent decades, increasing attention has turned to diet as a key modifiable factor in reproductive health. At the same time, dietary patterns worldwide have shifted markedly toward ultra-processed foods (UPFs), which are extensively processed and industrially manufactured products, typically high in added sugars, saturated and trans fats, salt, and additives, while low in fiber and essential nutrients (Monteiro *et al.*, 2010, 2019). These foods now account for up to 50–60% of daily energy intake in some high-income countries (Scrinis and Monteiro, 2022). In recognition of this concern, The Lancet recently released a landmark series emphasizing UPFs as a global threat, highlighting their role in non-communicable diseases, and advocating for international policy frameworks to limit UPF consumption (Baker *et al.*, 2025; Monteiro *et al.*, 2025; Scrinis *et al.*, 2025). While high maternal UPF consumption has been associated with adverse pregnancy and child outcomes, such as gestational diabetes, gestational hypertensive disorders, and increased adiposity in infancy, studies on the influence of maternal and paternal UPF intake on fertility and early development are extremely scarce (Zupo *et al.*, 2024; Morales-Suarez-Varela and Rocha-Velasco, 2025). To date, one study among 3060 American women has examined the association between UPF consumption and subfertility in women, reporting no association (Evans *et al.*, 2024), and one cohort study among 701 Dutch women with high-risk pregnancies demonstrated that higher periconceptional maternal UPF consumption was associated with smaller crown-rump length (CRL) during the first trimester (Smit *et al.*, 2022). Studies in both healthy and subfertile men have linked higher UPF consumption to poor semen quality (Lv *et al.*, 2022; Ceretti *et al.*, 2024; Valle-Hita *et al.*, 2024). Although both maternal and paternal health are known to influence reproductive success and development of offspring, no study has assessed the combined impact of parental UPF consumption on fecundability, subfertility, or early development. With UPF consumption rising rapidly worldwide, it is crucial to investigate how periconceptional UPF intake in both women and men affects fertility and the first developmental stages of pregnancy. Understanding these effects may guide evidence-based preconception care strategies to promote fertility, healthy pregnancy outcomes, and even long-term offspring health. We hypothesized that higher parental UPF intake during the periconceptional period is associated with lower fecundability, increased subfertility risk, and impaired embryonic and yolk sac development.

Therefore, in a population-based prospective cohort from the preconception period onwards, we first examined associations of periconceptional UPF consumption in women and men with fecundability and subfertility. Second, we explored whether periconceptional UPF consumption in women and men is associated with embryonic growth and yolk sac development.

## Materials and methods

### Study population

This study was embedded in the Generation R *Next* Study, a population-based prospective cohort study from the preconception period onwards in Rotterdam, the Netherlands, and is part of the Generation R Study Programme (Kooijman *et al.*, 2016). The general aim of this study is to identify preconception and early-pregnancy determinants of fertility, embryonic development, and childhood outcomes. Women and their partners from the general population were eligible if they were ≥18 years old, residing in Rotterdam, and actively trying to conceive or already pregnant. Couples were included in the preconception period or during pregnancy between 2017 and 2021. Follow-up of parents and their offspring is still ongoing and planned until childhood. Although enrollment was aimed at inclusion during preconception or early pregnancy, enrollment was allowed until delivery. In total, 33.2% and 52.8% of all inclusions were during the preconception period or in the first trimester of pregnancy, respectively. In total, there were 4036 participant episodes from 3604 unique women, leading to 3577 pregnancies of 3200 unique women at the end of the study. The current study is conducted within a subgroup of the original population, as the food frequency questionnaire (FFQ) for dietary assessment was distributed to a randomly selected subgroup of couples during early pregnancy. At the time of dietary assessment, all women were pregnant. In total, 1054 unique women provided information on dietary intake. We selected two separate study samples for the fertility and first trimester development analyses, as shown in Supplementary Fig. S1. For the fertility analyses, 831 unique women provided information on time to pregnancy. For the embryonic growth analyses, 704 unique women had a known last menstrual period (LMP) to determine gestational age and data for at least one first trimester ultrasound measurement. Dietary intake information was available for 651 male partners in the fertility population and 537 male partners in the embryonic growth population. From a biological perspective, women are central to pregnancy, therefore, partners were only included when data of the women were available.

### Ethical approval and consent to participate

The conduct of this study was approved by the Medical Ethical Committee of the Erasmus Medical Center, University Medical Center, Rotterdam (MEC 2016-589, NL57828.078.16), and written informed consent was acquired from participating women and men.

### Periconceptional UPF consumption in women and men

Periconceptional dietary intake of women and men was assessed during early pregnancy at a median of 12^+3 ^weeks of gestation (95% range 10^+6^, 18^+3^) using the Dutch version of the semi-quantitative HEalthy LIfe in an Urban Setting (HELIUS) FFQ. This FFQ was developed at the Amsterdam University Medical Center (UMC), in collaboration with the National Institute for Public Health and the Environment (RIVM) and Wageningen University (Beukers *et al.*, 2015), and its relative validity has been shown to range from acceptable to good (Visser *et al.*, 2020). Moreover, the performance of the HELIUS FFQ was comparable to 24-h dietary recall. Detailed information regarding the processing of the FFQ data has been described previously (Schipper *et al.*, 2024). Briefly, women and men were asked to report frequency and typical quantity consumed of >200 food items over the past 4 weeks. To improve accuracy in portion size reporting, additional questions addressed portion sizes through standardized units, household measures, or visual aids, such as colored photographs depicting various portion sizes. Each food item was linked to data from the National Institute for Public Health and the Netherlands Nutrition Centre (2011) to calculate average daily energy and nutrient intake (National Institute for Public Health and the Netherlands Nutrition Centre, 2011). Participants with implausible habitual daily energy intake, defined as <500 or >3500 kcal for women and <800 or >4000 kcal for men, were excluded from the analyses to correct for potential under- or overreporting.

Next, two University Medical Centers independently classified all food items according to the NOVA classification as non-UPFs or UPFs (NOVA group 4). For food items consisting of more than a single food, we calculated the UPF content of that food by weighting the individual (underlying) foods according to their frequency of consumption based on Dutch food consumption data (Beukers *et al.*, 2015). For instance, the food item ‘grains for porridge’ comprises four underlying foods; 79.7% breakfast product Brinta, 16.4% oatmeal, 2.3% Breakfast product Albona 7-grains-energy breakfast, and 1.6% rice flour Bambix. Only rice flour Bambix was classified as ultra-processed. Hence, the UPF content for the FFQ item ‘grains for porridge’ was 1.6%. Initial classification was performed by three researchers from Amsterdam UMC, with verification by two researchers from the Erasmus MC. Food assessment was based on definitions and examples by Monteiro *et al.* (2010, 2019). In case of uncertainty, websites of Dutch supermarkets were consulted to evaluate the ingredient lists. Disagreements were resolved by discussion until consensus was reached.

After classification, daily UPF consumption (in grams) was calculated by multiplying the UPF content of each food item by its reported consumption and expressed as a percentage of total food intake (in grams). Standard deviation scores (SDS) were calculated to examine associations continuously across the full distribution of UPF intake, allowing detection of small effects relevant on population level (referred to as ‘across the full range’). Furthermore, UPF consumption quartiles were created to simplify clinical applicability and examine potential threshold effects.

### Time to pregnancy

Time to pregnancy and mode of conception were assessed through questionnaires in preconception and early pregnancy. We used questions regarding the date at which women started trying to conceive and refrained from using contraceptives (start date actively pursuing pregnancy) and the start date of ART treatment, including intrauterine insemination, ovulation induction, IVF, and ICSI. The first day of the LMP was obtained from the obstetric caregiver, and confirmed during the ultrasound visits along with details on cycle regularity and average cycle duration. Time to pregnancy was calculated from the start date of actively pursuing pregnancy and the first day of the LMP. In women who achieved pregnancy using ART, we added 12 months to their time to pregnancy, as these treatments generally start after 1 year of not conceiving. Fecundability was defined as the probability of conceiving within 1 month. Time to pregnancy was categorized into two groups: <12 months (fertile) and ≥12 months (subfertile). Women who underwent ART were included in the subfertile group.

### First trimester embryonic and yolk sac development

For the growth analyses, we only included a subgroup of women with a known LMP and an average cycle length between 21 and 35 days. To determine gestational age, information on menstrual cycle and the LMP was obtained via questionnaires in early-pregnancy. During ultrasound visits, the first day of the LMP was confirmed, along with additional details on cycle regularity, average cycle duration, and use of ART. Trained sonographers performed 2D transvaginal ultrasound examinations of women in early pregnancy at median 7^+6 ^weeks (95% range 6^+6^, 8^+4^), median 9^+5 ^weeks (95% range 8^+5^, 10^+4^), and median 12^+2 ^weeks (95% range 10^+6^, 13^+3^) of gestation during visits to our dedicated research center, using a Philips^©^ Model Affiniti 70 or Model Epiq 7 equipped with a 3.0- to 9.0-MHz curved array transducer, or using a Voluson^©^ Model E10 equipped with a 4.5- to 11.9-MHz endocavity transducer or 4.0- to 9.0-MHz endocavity transducer, depending on which transducer effectuated the best imaging. Using the ultrasound data, we determined mean CRL as parameter for embryonic development, measured three times from the top of the fetal head to the buttocks in millimeters. The yolk sac was measured from outer edge to outer edge in two sagittal planes and one transverse plane to capture the length, width, and depth. These measurements were multiplied to determine the mean yolk sac volume. As the yolk sac typically degenerates around 10 weeks of gestation concurrent with placental development, the physiological meaning of yolk sac volume measured at 11 weeks remains poorly understood (Carter and Enders, 2016; Ross and Boroviak, 2020). While some animal studies have explored yolk sac diameter or volume (Pinter *et al.*, 1986; Ivanisević *et al.*, 2006; Dong *et al.*, 2016), there is scarcity of studies examining the course of yolk sac development across multiple time points. Given this limited evidence base, our analyses including yolk sac volume should be considered hypothesis generating. SDS were calculated for women with a reliable LMP and regular menstrual cycle using previously developed reference growth curves for CRL and yolk sac volume (unpublished data) using Generalized Additive Models of Location, Size, and Shape (GAMLSS) (Stasinopoulos and Rigby, 2008).

### Covariates

At enrollment, participants provided information on age, ethnicity, and highest education level via questionnaires. Ethnicity was determined based on the country of birth of both the participant and their parents. If a parent was born abroad, the participant was classified as of non-Dutch ethnic origin (Central Bureau for Statistics, Migrants in the Netherlands, 2003). Information on smoking, alcohol use, drug use, folic acid supplementation, parity, and any and daily nausea and vomiting were assessed through questionnaires filled in during the preconception period and/or in early pregnancy. Preconception BMI in women and BMI in men were assessed via clinical measurements of height and weight at enrollment and/or first trimester visits without shoes and heavy clothing, or obtained through questionnaires. BMI was categorized into underweight/normal weight (≤24.9 kg/m^2^) and overweight/obesity (≥25 kg/m^2^). Information on offspring sex was obtained from medical records or questionnaires.

### Statistical analysis

First, we performed a non-response analysis comparing the characteristics of women and men who were eligible for the FFQ and provided dietary intake data with those who were eligible and did not return the questionnaire, using *t*-tests, Mann–Whitney *U*-tests, chi-square tests, and Fisher’s Exact tests.

Second, we examined associations of UPFs consumed across the full range in SDS and quartiles with fecundability and subfertility in women and men using Cox proportional hazards regression models (R package *survival*) and logistic regression models, respectively. For the Cox proportional hazards model, the outcome was conception. The time variable was time to pregnancy (months/28 days). Details are provided in Supplementary materials and methods. The computed hazard ratios (HR) represent the fecundability ratios (FR). We specifically aimed to identify independent associations of UPF consumption in women and men on fertility and therefore examined associations in women and men singularly (referred to as separate models) and mutually adjusted for each other. To further investigate the attributions of UPF consumption among women and men, we categorized couples into low, discordant, and high UPF consumption groups, using a daily UPF consumption of 30% as a cut-off, which represents the mean UPF consumption in the Netherlands (Vellinga *et al.*, 2022). We first ran unadjusted models. Subsequently, models were adjusted for potential confounders, which were identified *a priori* through a literature search and utilization of a directed acyclic graph (Supplementary Fig. S2) (Axmon *et al.*, 2006; Zilaitiene *et al.*, 2007; Mook-Kanamori *et al.*, 2010; Rossi *et al.*, 2014; Olig *et al.*, 2019; van Duijn *et al.*, 2021; Pielage *et al.*, 2023; Graafland *et al.*, 2025). We considered the confounder-adjusted models as the main models. Models in women were adjusted for age, pre-pregnancy BMI, ethnicity, educational level, alcohol use, smoking, parity, and total energy intake. Models in men were adjusted for age, BMI, ethnicity, educational level, alcohol use, smoking, and total energy intake. Mutually adjusted models included both women’s and men’s UPF consumption and all of the above-mentioned covariates.

Third, we explored the associations of maternal UPF consumption in quartiles with longitudinal embryonic growth patterns throughout the first trimester using linear mixed models (R package *nlme*). UPF quartiles were included as main effects (intercepts) and as interaction term with gestational age to estimate their association with embryonic growth rates over time. The models included a random intercept and a random slope for gestational age at the individual level, allowing both baseline size and growth rate to vary between individuals. We performed *post hoc* analyses estimating marginal means of CRL at each gestational age by UPF quartiles. These models were unadjusted to estimate crude associations between maternal UPF intake and embryonic growth over time. As a second step, to examine associations between maternal UPF consumption and first trimester development in more detail, including the role of confounding, we analyzed associations of maternal UPF consumption in SDS and quartiles with CRL and yolk sac volume at 7, 9, and 11 weeks of gestation using linear regression models. Similarly, to assess the potential influence of paternal UPF intake, we explored the associations of paternal UPF consumption in SDS and quartiles with CRL and yolk sac volume at 7, 9, and 11 weeks using linear regression models. First trimester development models were adjusted for all covariates from the fertility models and additionally adjusted for menstrual cycle regularity and offspring sex. We performed a sensitivity analysis by repeating the analyses including women who experienced miscarriage or perinatal loss (n = 6), as UPF intake may influence the risk of pregnancy loss.

Missing values of covariates were imputed using Multiple Imputation by Chained Equations (R package *mice*) and pooled results were reported. The percentage of missing values varied from 0.0% to 13.3%. The statistical tests were two-sided and a significance level of *P*-value <0.05 was considered. All statistical analyses were performed using R version 4.4.1. (R Foundation for Statistical Computing, Vienna, Austria).

## Results

### Population and dietary characteristics

Population characteristics of the fertility study population are shown in Table 1. Median UPF consumption was 563 grams/day (IQR: 408, 816; 22.0% of total grams consumed) for women and 643 grams/day (IQR: 485, 866; 25.1% of total grams consumed) for men. Population characteristics for the embryonic growth study population are summarized in Supplementary Table S1, with 79.2% of women also included in the fertility group. The population characteristics were comparable. Supplementary Tables S2 and S3 present population characteristics stratified by UPF consumption in women and men. The correlation between a couple’s UPF intake was 0.34. Of the 651 couples, 200 couples (30.7%) had a low UPF intake, 371 couples (57.0%) showed discordant intake patterns, and 80 couples (12.3%) had a high UPF intake. Non-response analyses revealed that women and men with available data on total dietary intake were more likely to be of Dutch nationality, higher educated, nulliparous (in women), nonsmokers, and alcohol users, and have a lower (pre-pregnancy) BMI, compared to those without dietary intake data (Supplementary Table S4).

**Table 1.** Population characteristics.

	Women n = 831	Men n = 651
***Population characteristics***		
Age at dietary assessment (years), mean (SD)	32.1 (3.9)	34.2 (5.0)
Gestational age at dietary intake assessment (weeks^+ days^), median (95% range)	12^+3^ (10^+6^, 18^+3^)	12^+4^ (10^+6^, 21^+3^)
Ethnicity (n, %)		
Dutch	570 (68.6)	486 (74.7)
Other European	79 (9.5)	51 (7.8)
Non-European	178 (21.4)	111 (17.1)
Educational level, high (n, %)	675 (81.2)	487 (74.8)
(Pre-pregnancy) body mass index (kg/m^2^), median (IQR)	23.0 (21.1, 25.3)	24.6 (22.7, 26.8)
Overweight/obesity (n, %)	222 (26.7)	286 (43.9)
Smoking before pregnancy (n, %)	315 (37.9)	313 (48.0)
Alcohol use before pregnancy (n, %)	702 (84.5)	596 (91.6)
Drug use before pregnancy (n, %)	72 (8.7)	99 (15.2)
Periconceptional folic acid supplementation (n, %)	805 (96.9)	na
Parity, nulliparous (n, %)	595 (71.6)	na
Nausea and vomiting during early pregnancy (n, %)		
Any nausea	686 (82.6)	na
Any vomiting	212 (25.5)	na
Daily nausea and vomiting	20 (2.4)	na
Previous miscarriage (n, %)	142 (17.1)	na
Previously treated for a sexually transmitted disease (n, %)	170 (20.5)	na
Total energy intake (kcal/day), mean (SD)	1871.5 (501.0)	2349.4 (593.1)
Carbohydrate (grams/day), mean (SD)	215.4 (62.1)	250.2 (69.1)
Protein (grams/day), mean (SD)	74.5 (22.2)	95.7 (26.5)
Fat (grams/day), mean (SD)	68.3 (22.5)	88.0 (28.2)
Fiber (grams/day), mean (SD)	23 (6.9)	26.1 (8.3)
UPFs (grams/day), median (IQR)	563 (408, 816)	643 (485, 866)
% UPFs (of total grams consumed), median (IQR)	22.0 (15.7, 30.7)	25.1 (18.4, 33.0)
***Outcome characteristics***		
**Fertility outcomes**		
Time to pregnancy (months), median (IQR)	4.8 (1.2, 16.4)	na
Time to pregnancy ≥12 months or use of assisted reproductive technology (subfertility) (n, %)	254 (30.6)	na
Pregnancy as a result of fertility treatment (n, %)	108 (13.0)	na
**7-week ultrasound examination** *		
Gestational age (weeks^+ days^), median (95% range)	7^+6^ (6^+6^, 8^+4^)	na
Crown-rump length (mm), mean (SD)	14.0 (4.2)	na
Yolk sac volume (mm^3^), median (IQR)	127.2 (103.5, 150.7)	na
**9-week ultrasound examination** *		
Gestational age (weeks^+ days^), median (95% range)	9^+5^ (8^+5^, 10^+4^)	na
Crown-rump length (mm), mean (SD)	29.1 (6.5)	na
Yolk sac volume (mm^3^), median (IQR)	178.6 (148.7, 220.3)	na
**11-week ultrasound examination** *		
Gestational age (weeks^+ days^), median (95% range)	12^+2^ (10^+6^, 13^+3^)	na
Crown-rump length (mm), mean (SD)	59.9 (9.2)	na
Yolk sac volume (mm^3^), median (IQR)	208.1 (139.3, 293.1)	na

* Descriptives for ultrasound examinations were derived from smaller population for growth analysis (n = 704 women, see Supplementary Table S1).

IQR, interquartile range; na, not applicable; UPF, ultra-processed food.

### Parental UPF consumption and fertility

In women, no associations of UPF in quartiles or across the full range with fecundability were present (Table 2). For subfertility risk, women in the third quartile of UPF consumption had a higher subfertility risk as compared to women in the first quartile (*P*-value < 0.05), but no consistent linear trend across quartiles was visible. Higher UPF consumption across the full range tended to be associated with increased subfertility risk in women, but not statistically significant. Similar findings were present in the mutually adjusted models, considering UPF consumption of both women and men (Table 2).

**Table 2.** Associations of parental ultra-processed food (UPF) consumption with fecundability and subfertility risk.

	**Fecundability** ^a^	**Subfertility** ^b^
	Fecundability ratio (95% CI)	Odds ratio (95% CI)
**Women**	n = 831	
Percentage UPFs		
Quartile 1	*Reference*	*Reference*
Quartile 2	0.85 (0.70, 1.04)	1.54 (0.98, 2.42)
Quartile 3	0.85 (0.70, 1.04)	1.64 (1.03, 2.63)*
Quartile 4	0.96 (0.77, 1.18)	1.27 (0.78, 2.06)
Percentage UPFs (SDS)	0.97 (0.90, 1.05)	1.10 (0.94, 1.30)
**Men**	n = 651	
Percentage UPFs		
Quartile 1	*Reference*	*Reference*
Quartile 2	1.04 (0.83, 1.30)	0.94 (0.55, 1.60)
Quartile 3	0.95 (0.76, 1.20)	1.35 (0.80, 2.28)
Quartile 4	0.93 (0.73, 1.17)	1.69 (1.00, 2.85)*
Percentage UPFs (SDS)	0.92 (0.85, 0.99)*	1.37 (1.14, 1.65)*
**Couples** ^c^	n = 651	
**Women**		
Percentage UPFs		
Quartile 1	*Reference*	*Reference*
Quartile 2	0.90 (0.74, 1.10)	1.40 (0.88, 2.21)
Quartile 3	0.91 (0.74, 1.12)	1.34 (0.82, 2.18)
Quartile 4	1.12 (0.89, 1.41)	0.91 (0.54, 1.54)
Percentage UPFs (SDS)	1.01 (0.92, 1.11)	0.97 (0.77, 1.22)
**Men**		
Percentage UPFs		
Quartile 1	*Reference*	*Reference*
Quartile 2	1.01 (0.79, 1.29)	0.95 (0.54, 1.67)
Quartile 3	0.89 (0.68, 1.13)	1.37 (0.79, 2.36)
Quartile 4	0.86 (0.65, 1.14)	1.61 (0.92, 2.82)
Percentage UPFs (SDS)	0.90 (0.83, 0.99)*	1.36 (1.11, 1.67)*

^a^Values represent the fecundability per quartile increase (with quartile 1 as reference) or per standard deviation score (SDS) increase in the dietary share of UPFs for women and men. Fecundability represents the probability of conceiving within 1 month (28 days). Models were analyzed using Cox proportional hazards models. Fecundability ratios were derived from the hazard ratios of the Cox proportional hazards models.

^b^Values represent the odds of subfertility (≥12 months to conceive or use of assisted reproductive technology) per quartile increase (with quartile 1 as reference) or per standard deviation score (SDS) increase in the dietary share of UPFs for women and men. Models were analyzed using logistic regression models.

^c^In couples, models were mutually adjusted for their shared socio-demographic and lifestyle factors, including partner’s dietary intake.

Models in women were adjusted for maternal age, ethnicity, educational level, alcohol use, smoking, pre-pregnancy body mass index, parity, and total energy intake. Models in men were adjusted for paternal age, ethnicity, educational level, alcohol use, smoking, body mass index, and total energy intake. The mutually adjusted models included all confounders listed for both women and men.

* *P*-value <0.05.

In men, in quartile analyses, a non-significant trend was observed between higher paternal UPF consumption quartiles and decreased fecundability. When assessed across the full range, higher UPF consumption was associated with decreased fecundability [FR: 0.92, 95% CI: 0.85, 0.99, per SDS increase in paternal UPF consumption]. Similarly, men in the highest UPF quartile had an increased risk of subfertility compared to those in the lowest quartile [OR: 1.69, 95% CI 1.00, 2.85, for men in the fourth UPF quartile compared to the lowest quartile]. Higher UPF consumption across the full range in men was associated with increased subfertility risk [OR: 1.37, 95% CI: 1.14, 1.65, per SDS increase in paternal UPF consumption] (Table 2). In the mutually adjusted continuous models, higher paternal UPF consumption remained associated with decreased fecundability and increased subfertility risk after adjusting for the couple’s shared socio-demographic and lifestyle factors and female partner’s UPF consumption (all *P*-values <0.05) (Table 2). No associations were present for the categorized analyses based on a couple’s UPF consumption (Supplementary Table S5).

### Parental UPF consumption and embryonic growth

**Figure 1.**
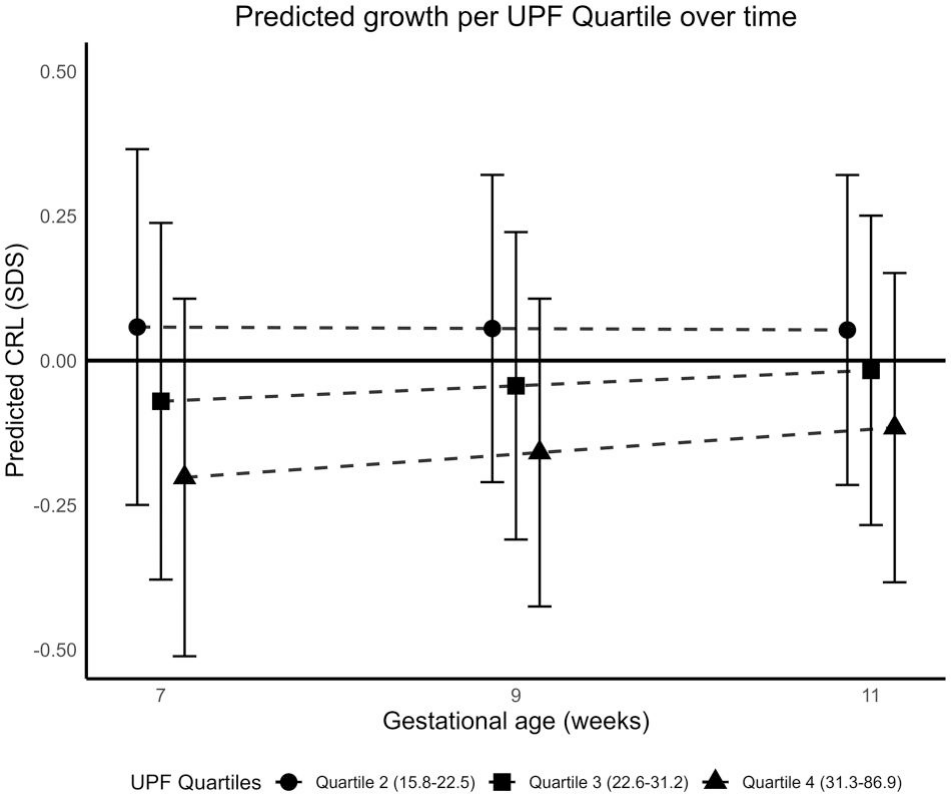
**First-trimester embryonic growth trajectories by maternal ultra-processed food (UPF) consumption quartiles.** Figure shows estimated embryonic growth trajectories based on standardized crown-rump length (CRL) at 7, 9, and 11 weeks gestation, with corresponding 95% confidence intervals, according to maternal UPF consumption quartiles. The lowest UPF quartile was used as the reference group (zero line). The legend displays UPF quartile ranges, representing the daily dietary share of UPFs. Estimates were derived from linear mixed models including random intercepts and slopes for gestational age. Trajectories reflect differences in standardized CRL for the upper three UPF quartiles compared to the lowest UPF quartiles.

Figure 1 shows embryonic growth patterns from 7 weeks of gestation onwards according to maternal UPF consumption quartiles. Compared to embryos of mothers in the lowest UPF quartile, embryos of mothers in the highest UPF quartile tended to be smaller throughout the first trimester of pregnancy, but this pattern was not significant. Regression coefficients for gestational age-independent (intercept) and gestational age-dependent differences (interaction) are given in Supplementary Table S6.

When examined separately at each first trimester ultrasound visit and adjusted for potential confounders, embryos of mothers in the highest UPF quartile tended to have a smaller CRL at 7 weeks of gestation compared to those in the lowest quartile, however, not statistically significant [Difference in CRL: −0.32 SDS, 95% CI −0.67, 0.03, for embryos of mothers in the fourth UPF quartile compared to the lowest quartile] (Table 3). A similar but weaker tendency was visible at 11 weeks, but not at 9 weeks gestation. Higher maternal UPF consumption across the full range was associated with a smaller CRL at 7 weeks of gestation [Difference in CRL: −0.13 SDS, 95% CI: −0.25, −0.01, per SDS increase in maternal UPF consumption]. These associations attenuated at 9 and 11 weeks gestation. Our results remained consistent when repeating the analyses including women who experienced miscarriage and perinatal loss (results not shown).

**Table 3.** Associations of parental ultra-processed food (UPF) consumption with crown-rump length.

	Crown-rump length (SDS)		
	95% CI		
**Women**	7 weeks gestation n = 294	9 weeks gestation n = 368	11 weeks gestation n = 647
Percentage UPFs			
Quartile 1	*Reference*	*Reference*	*Reference*
Quartile 2	0.10 (−0.22, 0.41)	−0.06 (−0.33, 0.22)	−0.05 (−0.26, 0.17)
Quartile 3	−0.04 (−0.39, 0.31)	−0.03 (−0.31, 0.26)	−0.07 (−0.30, 0.15)
Quartile 4	−0.32 (−0.67, 0.03)	0.00 (−0.29, 0.29)	−0.15 (−0.38, 0.09)
Percentage UPFs (SDS)	−0.13 (−0.25, −0.01)*	0.02 (−0.09, 0.12)	−0.06 (−0.14, 0.03)
**Men**	7 weeks gestation n = 227	9 weeks gestation n = 290	11 weeks gestation n = 493
Percentage UPFs			
Quartile 1	*Reference*	*Reference*	*Reference*
Quartile 2	0.31 (−0.04, 0.65)	0.33 (0.04, 0.61)*	0.20 (−0.04, 0.44)
Quartile 3	0.18 (−0.18, 0.54)	0.36 (0.06, 0.66)*	0.17 (−0.08, 0.41)
Quartile 4	0.20 (−0.14, 0.53)	0.07 (−0.22, 0.36)	0.14 (−0.12, 0.40)
Percentage UPFs (SDS)	0.02 (−0.10, 0.14)	0.02 (−0.08, 0.13)	0.05 (−0.05, 0.14)

Values represent regression coefficients (95% confidence intervals) from linear regression models that reflect differences in standard deviation score (SDS) of crown-rump length (CRL) per quartile (with quartile 1 as reference) or per SDS increase in the dietary share of UPFs for women and men.

Confounder models for women included maternal age, ethnicity, educational level, alcohol use, smoking, pre-pregnancy body mass index, total energy intake, parity, menstrual cycle regularity, and offspring sex. Confounder models for men included paternal age, ethnicity, educational level, alcohol use, smoking, body mass index, total energy intake, maternal parity, menstrual cycle regularity, and offspring sex.

* *P*-value < 0.05.

Embryos of fathers in the second and third UPF quartile had a larger CRL as compared to embryos in the lowest quartile at 9 weeks gestation, but not at 7 or 11 weeks gestation [Difference in CRL: 0.33 SDS, 95% CI: 0.04, 0.61; 0.36 SDS, 95% CI: 0.06, 0.66, for embryos of fathers in the second and third UPF quartile compared to the lowest quartile, respectively], and no consistent linear trend emerged across quartiles. Paternal UPF consumption across the full range was not associated with CRL (Table 3).

### Parental UPF consumption and yolk sac development

In quartile analyses, we observed a trend toward smaller yolk sac volume with increasing maternal UPF quartiles at 7 weeks gestation, with the strongest effect size observed for women in the fourth quartile as compared to the lowest quartile [Difference in yolk sac volume: −0.41 SDS, 95% CI: −0.75, −0.06, for women in the fourth UPF quartile as compared to the lowest quartile] (Table 4). No consistent pattern was observed at 9 and 11 weeks gestation. Higher maternal UPF consumption across the full range was associated with smaller yolk sac volume at 7 weeks of gestation [Difference in yolk sac volume: −0.14 SDS, 95% CI: −0.26, −0.02, per SDS increase in maternal UPF consumption], which attenuated at 9 and 11 weeks of gestation. Our results remained consistent when repeating the analyses including women who experienced miscarriage and perinatal loss (results not shown).

**Table 4.** Associations of parental ultra-processed food (UPF) consumption with yolk sac volume.

	Yolk sac volume (SDS)		
	95% CI		
**Women**	7 weeks gestation n = 289	9 weeks gestation n = 349	11 weeks gestation n = 104
Percentage UPFs			
Quartile 1	*Reference*	*Reference*	*Reference*
Quartile 2	−0.02 (−0.33, 0.29)	0.02 (−0.27, 0.31)	−0.33 (−0.74, 0.08)
Quartile 3	−0.24 (−0.57, 0.10)	0.02 (−0.29, 0.32)	−0.14 (−0.54, 0.27)
Quartile 4	−0.41 (−0.75, −0.06)*	−0.05 (−0.37, 0.26)	−0.06 (−0.46, 0.33)
Percentage UPFs (SDS)	−0.14 (−0.26, −0.02)*	0.01 (−0.10, 0.12)	−0.05 (−0.20, 0.10)
**Men**	7 weeks gestation n = 223	9 weeks gestation n = 277	11 weeks gestation n = 83
Percentage UPFs			
Quartile 1	*Reference*	*Reference*	*Reference*
Quartile 2	0.25 (−0.10, 0.60)	0.02 (−0.29, 0.33)	−0.11 (−0.59, 0.37)
Quartile 3	0.29 (−0.08, 0.66)	0.39 (0.06, 0.72)*	−0.11 (−0.62, 0.39)
Quartile 4	0.26 (−0.08, 0.60)	0.11 (−0.21, 0.43)	−0.06 (−0.57, 0.44)
Percentage UPFs (SDS)	0.07 (−0.05, 0.19)	0.06 (−0.05, 0.17)	−0.02 (−0.21, 0.17)

Values represent regression coefficients (95% confidence intervals) from linear regression models that reflect differences in standard deviation score (SDS) of yolk sac volume per quartile (with quartile 1 as reference) or per SDS increase in the dietary share of UPFs for women and men.

Models in women were adjusted for maternal age, ethnicity, educational level, alcohol use, smoking, pre-pregnancy body mass index, total energy intake, parity, menstrual cycle regularity, and offspring sex. Models in men were adjusted for paternal age, ethnicity, educational level, alcohol use, smoking, body mass index, total energy intake, maternal parity, menstrual cycle regularity, and offspring sex.

* *P*-value < 0.05.

In men, no consistent associations were observed between UPF consumption and yolk sac volume, either in quartile analyses or in the continuous analyses (Table 4).

## Discussion

### Principal findings

In this population-based prospective cohort study from preconception onwards, we observed that higher periconceptional UPF consumption in women was not consistently associated with fertility outcomes, but was across the full range associated with smaller first trimester CRL and yolk sac volume, especially early in pregnancy. In contrast, higher UPF consumption in men was across the full range associated with decreased fecundability and an increased risk of subfertility, but not consistently with first trimester embryonic and yolk sac development.

### Comparison with existing literature

With UPFs accounting for a growing proportion of daily energy intake worldwide, understanding their potential consequences for reproductive health and embryonic development is becoming increasingly important (Monteiro *et al.*, 2025). Research on the impact of UPF consumption on reproductive health in women is limited, with only one cross-sectional study to date examining UPF consumption and reproductive health in females. This study, including 3060 American women of reproductive age, found no association between UPF consumption and subfertility risk (Evans *et al.*, 2024). Importantly, they solely focused on women’s UPF consumption and did not take into consideration partner’s diet or other lifestyle factors. While not directly addressing UPFs, a multi-center pregnancy-based cohort study including 5598 nulliparous women found that high fast food intake (>4 times/week compared to none), often rich in UPFs, was associated with increased time to pregnancy and infertility (Grieger *et al.*, 2014). Our study is the first to investigate the association between UPF consumption, fecundability, and subfertility in women and men of the general population, considering independent and combined contributions. We found no association between UPF consumption and fertility outcomes in women, although we observed a non-significant trend toward increased subfertility risk with higher UPF consumption. Taken together with prior research, our findings suggest that the impact of UPFs on reproductive health in women may depend less on overall UPF consumption and more on specific subgroups of UPFs. For instance, certain subgroups of UPFs, such as artificially sweetened beverages and processed meats, have been associated with an increased risk of multimorbidity, while other subgroups, like ultra-processed breads and cereals, showed no such association (Cordova *et al.*, 2023). In our study population, dietary patterns were relatively healthy, with non-UPFs and drinks comprising a large proportion of intake. Among the UPFs consumed, processed breads were especially common. This limited variability in UPF intake and type of UPF products may have reduced our ability to detect an association with fertility outcomes in women.

Research on UPF consumption and male fertility has been limited to studies examining semen quality parameters. One hospital-based case-control study (549 cases and 581 controls) reported a positive association between UPF consumption and odds of asthenozoospermia (Lv *et al.*, 2022). Similarly, two cross-sectional studies among young and healthy Mediterranean men showed an inverse association between UPF consumption with sperm count, concentration, and motility (Ceretti *et al.*, 2024; Valle-Hita *et al.*, 2024). We showed for the first time that higher UPF consumption was associated with reduced fecundability and increased subfertility risk in men, independent of socio-demographic and lifestyle factors, as well as their female partners’ UPF consumption. The magnitude of the association was strongest for males within the highest UPF quartile, but also present across the full range when assessed in continuous analyses. This association may be explained by the sensitivity of sperm to dietary composition, which has been shown to influence sperm motility and overall male fertility (Nätt *et al.*, 2019). Couples’ UPF consumption was not linked to fecundability, though couples with high intake of UPF by both partners tended to be more subfertile than those with low intake. These analyses were constrained by low numbers of couples with high UPFs, which may have limited the ability to detect associations. Taken together, our findings illustrate that higher paternal UPF consumption was independently associated with lower fecundability and higher subfertility risk, while no consistent associations were observed in women, indicating that paternal diet quality may be an important yet underrecognized target for preconception care and fertility interventions. Notably, these associations were observed even at relatively low levels of UPF intake, which may suggest that modest dietary improvements in men could have meaningful benefits for reproductive outcomes.

The first trimester encompasses multiple critical stages, including morphogenesis and organogenesis, essential for embryonic growth and yolk sac development, making it highly sensitive to external exposures. Impaired first trimester growth has been associated with adverse birth outcomes, such as preterm birth and low birth weight, as well as an unfavorable cardiovascular profile in childhood (Mook-Kanamori *et al.*, 2010; Jaddoe *et al.*, 2014). During these early weeks, the yolk sac is an essential structure for embryonic development, supporting hematopoiesis, vasculogenesis, and nutrient supply to the embryo until the placenta is sufficiently developed by ∼12 weeks gestation (Ross and Boroviak, 2020; Shibata *et al.*, 2023). A study among 122 women previously reported that yolk sac size significantly impacts embryonic growth in the first trimester, underlining the importance of the yolk sac as an early marker of embryonic growth (Odland Karlsen *et al.*, 2019). Little is known about yolk sac development during the first trimester, particularly regarding the physiological significance of yolk sac presence at 11 weeks, which could reflect a normal biological adaptation or an underlying abnormality in placental development (Tan *et al.*, 2012). To date, one Dutch tertiary hospital-based prospective cohort study among 701 women reported that maternal periconceptional UPF consumption was associated with smaller growth trajectories of CRL and embryonic volume between 7 and 11 weeks of gestation (Smit *et al.*, 2022). In line with findings from the hospital-based Dutch cohort study, we found that higher maternal UPF consumption was associated with smaller early embryonic growth among women from the general population, and these associations weakened across the first trimester of pregnancy. Higher maternal UPF consumption was also associated with a smaller yolk sac volume in the first trimester, potentially indicating impaired nutrient availability to the embryo, which may contribute to restricted embryonic growth in the earliest stage of pregnancy. Whether these early embryonic growth alterations are related to subsequent effects on birth outcomes and long-term offspring health outcomes needs to be further studied. Despite growing evidence suggesting that paternal diet may influence early embryonic development through epigenetic and sperm-mediated mechanisms (Soubry, 2018), we observed no consistent associations between paternal UPF consumption and parameters of embryonic and yolk sac development. This may indicate that direct maternal intrauterine influences have a stronger impact on first-trimester development than potential epigenetic effects related to reduced semen quality.

Our findings highlight distinct, sex-specific associations of maternal and paternal UPF consumption with fertility and embryonic development. The associations of higher UPF consumption with lower fecundability in men and reduced embryonic growth in women may be explained by the low nutritional value of UPFs. Adequate nutrition is crucial for semen quality, such as sperm production, motility, and integrity (Pecora *et al.*, 2023). Poor nutrition may elevate oxidative stress and testosterone levels, potentially affecting mitochondria, essential organelles that sustain sperm function (Ferramosca and Zara, 2022). In addition, UPFs are frequently packaged in materials containing endocrine-disrupting chemicals, such as phthalates and bisphenols (Buckley *et al.*, 2019; Baker *et al.*, 2024). These substances may alter hormone levels, potentially affecting male fertility. During pregnancy, higher maternal UPF consumption may reduce nutrient availability to the developing embryo. Yolk sac genetic expression may be sensitive to nutrient availability, such as metals and vitamins (White *et al.*, 2024). These genes are essential for biosynthesis and nutrient transport to the embryo, regulating embryonic development. Impairment of the yolk sac’s ability to provide nutrients to the embryo may explain the smaller CRL observed with higher maternal UPF consumption. A second hypothesis is the increased levels of oxidative stress linked with higher UPF consumption, which could disrupt redox homeostasis (Edalati *et al.*, 2021; Swain *et al.*, 2022; Divvela *et al.*, 2025). This imbalance may result in damage to biomolecules, such as lipids or proteins, possibly adversely impacting embryonic and yolk sac development.

Our findings are important from both an etiological and public health perspective, given the high and rising consumption of UPFs worldwide. As parental UPF consumption was not only associated with fertility but also with first-trimester embryonic growth, our research highlights the need for greater awareness of the potential consequences of poor diet quality and support the inclusion of both partners in preconception care and dietary counseling. Notably, the median share of UPFs of the diet in our study population was relatively low compared to averages in other high-income countries, which can reach up to 60%. In populations with higher UPF intake, the adverse influences on fertility and early development may even be more pronounced. As we observed associations across the full range of UPF consumption, even modest reductions in UPF intake may have beneficial impacts. Investigating UPFs as a whole is valuable, as diet is shaped by overall eating habits rather than single food groups (Hu, 2002). The UPF classification is an effective tool for dietary behavior, as they reflect wide-ranging recognizable patterns of food habits, offering a practical framing for dietary guidance (Scrinis and Monteiro, 2022). Both studies involving specific types of UPF products and UPFs are crucial to develop comprehensive and effective dietary guidelines (Tapsell *et al.*, 2016). These studies should assess maternal and paternal dietary intake at multiple time points during preconception and pregnancy to identify sensitive windows of exposure and subsequent consequences for fetal and childhood development. Ideally, these studies should use a randomized controlled trial design to assess causality and include a larger variability in the percentage of UPFs consumed among diverse multi-ethnic populations. These randomized controlled trials should focus on reducing overall UPF consumption and specific types of UPFs in couples through education on UPFs and support for making dietary changes, for example by psychological support to increase self-efficacy or by healthy food vouchers to reduce concerns about costs. Policy changes in line with the recommendations from The Lancet series on UPFs, such as UPF labeling, taxation, and marketing restrictions, may shift population exposure of UPFs, providing quasi-experimental evidence of impact (Baker *et al.*, 2025). Finally, these studies should be used to generate robust clinical guidelines in which the importance of diet composition with respect to reproduction and embryonic development is discussed.

### Strengths and limitations

Strengths of our study are the prospective study design from preconception onwards, large sample size, and repeatedly measured embryonic growth and yolk sac data throughout early-pregnancy. Non-response bias and the selection towards a relatively healthy Dutch population may have affected the generalizability of our findings and led to reduced statistical power. We collected dietary intake data at one time point during the first trimester due to the design of our study and considering the study burden for participants, serving as a proxy for periconceptional diet. Our approach was supported by existing literature, which shows that dietary patterns generally remain stable from the preconceptional period to early pregnancy (Cucó *et al.*, 2006; Crozier *et al.*, 2009). As we collected dietary data in early pregnancy, only couples who became pregnant are included in our study sample. In this study population, 30.6% of women experienced subfertility, which is higher as compared to population figures in the Netherlands (Peters *et al.*, 2023). This suggests that couples with fertility concerns may have been more likely to participate, leading to a disproportionate representation. The inclusion of women conditional on their pregnancy status could introduce selection bias in the fertility analyses. We expect that this would likely result in an underestimation of the effect estimates, as associations of reduced fecundability and increased subfertility were already evident within our current study population. Further studies are needed to replicate our findings in diverse study populations with repeated dietary data collection during preconception and pregnancy. Our study population consumed fewer UPFs on average compared to the Dutch population (26.4% vs 30.3%, respectively) (Vellinga *et al.*, 2022). Although FFQs are widely used in population-based cohorts for their ability to capture long-term dietary patterns with minimal participant burden, they may be less accurate in estimating absolute intake and are subject to measurement error and reporting bias. Due to the data processing of the dietary data, we could not determine the amount of UPFs consumed in kilocalories/day. However, by expressing UPF consumption in grams/day, we could account for UPFs with zero caloric value. Despite adjusting for various confounders, residual confounding, for example by physical activity, could still be present, as in any observational study.

## Conclusions

Among couples in the general population, we demonstrated sex-specific associations between periconceptional UPF consumption, reproductive outcomes, and early embryonic development. Higher maternal UPF consumption was associated with smaller early embryonic growth and yolk sac volume in the first trimester of pregnancy, while higher paternal UPF intake was associated with reduced fecundability and increased subfertility risk. These findings highlight the importance of considering dietary habits of both partners in preconception care, which may offer a valuable opportunity to support conception and optimize early developmental trajectories.

## Supplementary Material

deag023_Supplementary_materials_and_methods

deag023_Supplementary_Figure_S1

deag023_Supplementary_Figure_S2

deag023_Supplementary_Table_S1

deag023_Supplementary_Table_S2

deag023_Supplementary_Table_S3

deag023_Supplementary_Table_S4

deag023_Supplementary_Table_S5

deag023_Supplementary_Table_S6

## Data Availability

The datasets generated during and/or analyzed during the current study are not publicly available due to reasons of sensitivity but are available from the corresponding author upon reasonable request.

## References

[deag023-B1] Axmon A , Rylander L, Albin M, Hagmar L. Factors affecting time to pregnancy. Hum Reprod 2006;21:1279–1284. 16410331 10.1093/humrep/dei469

[deag023-B2] Baker BH , Melough MM, Paquette AG, Barrett ES, Day DB, Kannan K, Hn Nguyen R, Bush NR, LeWinn KZ, Carroll KN et al Ultra-processed and fast food consumption, exposure to phthalates during pregnancy, and socioeconomic disparities in phthalate exposures. Environ Int 2024;183:108427. 38194756 10.1016/j.envint.2024.108427 PMC10834835

[deag023-B3] Baker P , Slater S, White M, Wood B, Contreras A, Corvalán C, Gupta A, Hofman K, Kruger P, Laar A et al Towards unified global action on ultra-processed foods: understanding commercial determinants, countering corporate power, and mobilising a public health response. Lancet 2025;406:2703–2726. 41270764 10.1016/S0140-6736(25)01567-3

[deag023-B4] Barker DJ. The origins of the developmental origins theory. J Intern Med 2007;261:412–417. 17444880 10.1111/j.1365-2796.2007.01809.x

[deag023-B5] Beukers MH , Dekker LH, de Boer EJ, Perenboom CW, Meijboom S, Nicolaou M, de Vries JH, Brants HA. Development of the HELIUS food frequency questionnaires: ethnic-specific questionnaires to assess the diet of a multiethnic population in The Netherlands. Eur J Clin Nutr 2015;69:579–584. 25226823 10.1038/ejcn.2014.180

[deag023-B6] Buckley JP , Kim H, Wong E, Rebholz CM. Ultra-processed food consumption and exposure to phthalates and bisphenols in the US National Health and Nutrition Examination Survey, 2013-2014. Environ Int 2019;131:105057. 31398592 10.1016/j.envint.2019.105057 PMC6728187

[deag023-B7] Carter AM , Enders AC. Placentation in mammals: definitive placenta, yolk sac, and paraplacenta. Theriogenology 2016;86:278–287. 27155730 10.1016/j.theriogenology.2016.04.041

[deag023-B8] Central Bureau for Statistics. *Migrants in the Netherlands*. Voorburg/Heerlen, the Netherlands: Central Bureau for Statistics, 2003.

[deag023-B9] Ceretti E , Bonaccio M, Iacoviello L, Di Castelnuovo A, Ruggiero E, Donato F, Lorenzetti S, Zani D, Montano L. Consumption of ultra-processed foods and semen quality in healthy young men living in Italy. Nutrients 2024;16:4129. 39683523 10.3390/nu16234129 PMC11644553

[deag023-B10] Cordova R , Viallon V, Fontvieille E, Peruchet-Noray L, Jansana A, Wagner KH, Kyrø C, Tjønneland A, Katzke V, Bajracharya R et al Consumption of ultra-processed foods and risk of multimorbidity of cancer and cardiometabolic diseases: a multinational cohort study. Lancet Reg Health Eur 2023;35:100771. 38115963 10.1016/j.lanepe.2023.100771 PMC10730313

[deag023-B11] Cox CM , Thoma ME, Tchangalova N, Mburu G, Bornstein MJ, Johnson CL, Kiarie J. Infertility prevalence and the methods of estimation from 1990 to 2021: a systematic review and meta-analysis. Hum Reprod Open 2022;2022:hoac051. 36483694 10.1093/hropen/hoac051 PMC9725182

[deag023-B12] Crozier SR , Robinson SM, Godfrey KM, Cooper C, Inskip HM. Women’s dietary patterns change little from before to during pregnancy. J Nutr 2009;139:1956–1963. 19710161 10.3945/jn.109.109579 PMC3113465

[deag023-B13] Cucó G , Fernández-Ballart J, Sala J, Viladrich C, Iranzo R, Vila J, Arija V. Dietary patterns and associated lifestyles in preconception, pregnancy and postpartum. Eur J Clin Nutr 2006;60:364–371. 16340954 10.1038/sj.ejcn.1602324

[deag023-B15] Divvela SSK , Gallorini M, Gellisch M, Patel GD, Saso L, Brand-Saberi B. Navigating redox imbalance: the role of oxidative stress in embryonic development and long-term health outcomes. Front Cell Dev Biol 2025;13:1521336. 40206404 10.3389/fcell.2025.1521336 PMC11979171

[deag023-B16] Dong D , Reece EA, Lin X, Wu Y, AriasVillela N, Yang P. New development of the yolk sac theory in diabetic embryopathy: molecular mechanism and link to structural birth defects. Am J Obstet Gynecol 2016;214:192–202. 26432466 10.1016/j.ajog.2015.09.082 PMC4744545

[deag023-B17] Donovan MF , Arbor TC, Bordoni B. Embryology, Yolk Sac. Treasure Island, Florida, USA: StatPearls Publishing, 2025. 32310425

[deag023-B19] Edalati S , Bagherzadeh F, Asghari Jafarabadi M, Ebrahimi-Mamaghani M. Higher ultra-processed food intake is associated with higher DNA damage in healthy adolescents. Br J Nutr 2021;125:568–576. 32513316 10.1017/S0007114520001981

[deag023-B20] Evans AT , Alur-Gupta S, Knutson AJ, Martinez Steele E, Meng Y, Vitek WS. Consumption of ultra-processed foods is not associated with female infertility: a cross-sectional study of the National Health and Nutrition Examination Survey (NHANES), 2013-2018. Fertil Steril 2024;122:e121.

[deag023-B21] Ferramosca A , Zara V. Diet and male fertility: the impact of nutrients and antioxidants on sperm energetic metabolism. Int J Mol Sci 2022;23:2542. 35269682 10.3390/ijms23052542 PMC8910394

[deag023-B22] Graafland N , Rousian M, de Zwart ML, Steegers-Theunissen RPM, Steegers EAP, Posthumus AG. Parental conditions, modifiable lifestyle factors, and first trimester growth and development: a systematic review. Hum Reprod Update 2025;31:166–182. 39953705 10.1093/humupd/dmaf001 PMC12046076

[deag023-B23] Grieger JA , Grzeskowiak LE, Clifton VL. Preconception dietary patterns in human pregnancies are associated with preterm delivery. J Nutr 2014;144:1075–1080. 24790026 10.3945/jn.114.190686

[deag023-B24] Hoffman DJ , Reynolds RM, Hardy DB. Developmental origins of health and disease: current knowledge and potential mechanisms. Nutr Rev 2017;75:951–970. 29186623 10.1093/nutrit/nux053

[deag023-B25] Hu FB. Dietary pattern analysis: a new direction in nutritional epidemiology. Curr Opin Lipidol 2002;13:3–9. 11790957 10.1097/00041433-200202000-00002

[deag023-B26] Indrio F , Pietrobelli A, Dargenio VN, Marchese F, Grillo A, Vural M, Giardino I, Pettoello-Mantovani M. The key 1000 life-changing days. Glob Pediatr 2023;4:100049.

[deag023-B27] Ivanisević M , Djelmis J, Jalsovec D, Bljajic D. Ultrasonic morphological characteristics of yolk sac in pregnancy complicated with type-1 diabetes mellitus. Gynecol Obstet Invest 2006;61:80–86. 16224187 10.1159/000088933

[deag023-B28] Jaddoe VW , de Jonge LL, Hofman A, Franco OH, Steegers EA, Gaillard R. First trimester fetal growth restriction and cardiovascular risk factors in school age children: population based cohort study. BMJ 2014;348:g14. 24458585 10.1136/bmj.g14 PMC3901421

[deag023-B29] Kooijman MN , Kruithof CJ, van Duijn CM, Duijts L, Franco OH, van IMH, de Jongste JC, Klaver CC, van der Lugt A, Mackenbach JP et al The Generation R Study: design and cohort update 2017. Eur J Epidemiol 2016;31:1243–1264. 28070760 10.1007/s10654-016-0224-9 PMC5233749

[deag023-B30] Lv JL , Wu QJ, Wang XB, Du Q, Liu FH, Guo RH, Leng X, Pan BC, Zhao YH. Intake of ultra-processed foods and asthenozoospermia odds: a hospital-based case-control study. Front Nutr 2022;9:941745. 36337657 10.3389/fnut.2022.941745 PMC9630735

[deag023-B31] Monteiro CA , Cannon G, Levy RB, Moubarac JC, Louzada ML, Rauber F, Khandpur N, Cediel G, Neri D, Martinez-Steele E et al Ultra-processed foods: what they are and how to identify them. Public Health Nutr 2019;22:936–941. 30744710 10.1017/S1368980018003762 PMC10260459

[deag023-B32] Monteiro CA , Levy RB, Claro RM, Castro IR, Cannon G. A new classification of foods based on the extent and purpose of their processing. Cad Saude Publica 2010;26:2039–2049. 21180977 10.1590/s0102-311x2010001100005

[deag023-B33] Monteiro CA , Louzada ML, Steele-Martinez E, Cannon G, Andrade GC, Baker P, Bes-Rastrollo M, Bonaccio M, Gearhardt AN, Khandpur N et al Ultra-processed foods and human health: the main thesis and the evidence. Lancet 2025;406:2667–2684. 41270766 10.1016/S0140-6736(25)01565-X

[deag023-B34] Mook-Kanamori DO , Steegers EA, Eilers PH, Raat H, Hofman A, Jaddoe VW. Risk factors and outcomes associated with first-trimester fetal growth restriction. Jama 2010;303:527–534. 20145229 10.1001/jama.2010.78

[deag023-B35] Morales-Suarez-Varela M , Rocha-Velasco OA. Impact of ultra-processed food consumption during pregnancy on maternal and child health outcomes: a comprehensive narrative review of the past five years. Clin Nutr ESPEN 2025;65:288–304. 39662587 10.1016/j.clnesp.2024.12.006

[deag023-B18] National Institute for Public Health and the Netherlands Nutrition Centre. *Dutch Food Composition Table 2011*. The Hague, the Netherlands: National Institute for Public Health and the Netherlands Nutrition Centre, 2011.

[deag023-B36] Nätt D , Kugelberg U, Casas E, Nedstrand E, Zalavary S, Henriksson P, Nijm C, Jäderquist J, Sandborg J, Flinke E et al Human sperm displays rapid responses to diet. PLoS Biol 2019;17:e3000559. 31877125 10.1371/journal.pbio.3000559 PMC6932762

[deag023-B37] Odland Karlsen H , Johnsen SL, Rasmussen S, Trae G, Reistad HMT, Kiserud T. The human yolk sac size reflects involvement in embryonic and fetal growth regulation. Acta Obstet Gynecol Scand 2019;98:176–182. 30218536 10.1111/aogs.13466

[deag023-B38] Olig E , Mountan S, Beal JR, Sahmoun AE. Race and length of time pursuing pregnancy among women who utilized medical help to get pregnant. J Family Reprod Health 2019;13:146–153. 32201489 PMC7072030

[deag023-B39] Pecora G , Sciarra F, Gangitano E, Venneri MA. How food choices impact on male fertility. Curr Nutr Rep 2023;12:864–876. 37861951 10.1007/s13668-023-00503-x PMC10766669

[deag023-B40] Peters LL , Groen H, Sijtsma A, Jansen D, Hoek A. Women of reproductive age living in the North of the Netherlands: Lifelines Reproductive Origins of Adult Health and Disease (Lifelines-ROAHD) cohort. BMJ Open 2023;13:e063890. 10.1136/bmjopen-2022-063890 PMC10186082 37169493

[deag023-B41] Pielage M , El Marroun H, Odendaal HJ, Willemsen SP, Hillegers MHJ, Steegers EAP, Rousian M. Alcohol exposure before and during pregnancy is associated with reduced fetal growth: the Safe Passage Study. BMC Med 2023;21:318. 37612658 10.1186/s12916-023-03020-4 PMC10463675

[deag023-B42] Pinter E , Reece EA, Leranth CZ, Sanyal MK, Hobbins JC, Mahoney MJ, Naftolin F. Yolk sac failure in embryopathy due to hyperglycemia: ultrastructural analysis of yolk sac differentiation associated with embryopathy in rat conceptuses under hyperglycemic conditions. Teratology 1986;33:73–84. 3738811 10.1002/tera.1420330110

[deag023-B43] Ross C , Boroviak TE. Origin and function of the yolk sac in primate embryogenesis. Nat Commun 2020;11:3760. 32724077 10.1038/s41467-020-17575-w PMC7387521

[deag023-B44] Rossi BV , Abusief M, Missmer SA. Modifiable risk factors and infertility: what are the connections? Am J Lifestyle Med 2014;10:220–231. 27594813 10.1177/1559827614558020 PMC5007064

[deag023-B45] Schipper MC , Boxem AJ, Blaauwendraad SM, Mulders A, Jaddoe VWV, Gaillard R. Associations of periconception dietary glycemic index and load with fertility in women and men: a study among couples in the general population. BMC Med 2024;22:499. 39468525 10.1186/s12916-024-03718-z PMC11520767

[deag023-B46] Scrinis G , Monteiro C. From ultra-processed foods to ultra-processed dietary patterns. Nat Food 2022;3:671–673. 37118150 10.1038/s43016-022-00599-4

[deag023-B47] Scrinis G , Popkin BM, Corvalan C, Duran AC, Nestle M, Lawrence M, Baker P, Monteiro CA, Millett C, Moubarac JC et al Policies to halt and reverse the rise in ultra-processed food production, marketing, and consumption. Lancet 2025;406:2685–2702. 41270767 10.1016/S0140-6736(25)01566-1

[deag023-B48] Shibata M , Makihara N, Iwasawa A. The yolk sac’s essential role in embryonic development. Rev Agric Sci 2023;11:243–258.

[deag023-B49] Smit AJP , Hojeij B, Rousian M, Schoenmakers S, Willemsen SP, Steegers-Theunissen RPM, van Rossem L. A high periconceptional maternal ultra-processed food consumption impairs embryonic growth: the Rotterdam Periconceptional Cohort. Clin Nutr 2022;41:1667–1675. 35772220 10.1016/j.clnu.2022.06.006

[deag023-B50] Soubry A. POHaD: why we should study future fathers. Environ Epigenet 2018;4:dvy007. 29732171 10.1093/eep/dvy007 PMC5920283

[deag023-B51] Stasinopoulos DM , Rigby RA. Generalized additive models for location scale and shape (GAMLSS) in R. J Stat Soft 2008;23:1–46.

[deag023-B52] Sun H , Gong TT, Jiang YT, Zhang S, Zhao YH, Wu QJ. Global, regional, and national prevalence and disability-adjusted life-years for infertility in 195 countries and territories, 1990-2017: results from a global burden of disease study, 2017. Aging (Albany NY) 2019;11:10952–10991. 31790362 10.18632/aging.102497 PMC6932903

[deag023-B53] Swain N , Moharana AK, Jena SR, Samanta L. Impact of oxidative stress on embryogenesis and fetal development. Adv Exp Med Biol 2022;1391:221–241. 36472825 10.1007/978-3-031-12966-7_13

[deag023-B54] Tan S , Pektas MK, Ozcan AS, Akçay Y, Ozat M, Arslan H. Frequency of a persistent yolk sac and its relationship with the gestational outcome. J Ultrasound Med 2012;31:697–702. 22535716 10.7863/jum.2012.31.5.697

[deag023-B55] Tapsell LC , Neale EP, Satija A, Hu FB. Foods, nutrients, and dietary patterns: interconnections and implications for dietary guidelines. Adv Nutr 2016;7:445–454. 27184272 10.3945/an.115.011718 PMC4863273

[deag023-B56] Taylor A. ABC of subfertility: extent of the problem. BMJ 2003;327:434–436. 12933733 10.1136/bmj.327.7412.434 PMC188498

[deag023-B57] Valle-Hita C , Salas-Huetos A, Fernández de la Puente M, Martínez M, Canudas S, Palau-Galindo A, Mestres C, Manzanares JM, Murphy MM, Marquès M et al Ultra-processed food consumption and semen quality parameters in the Led-Fertyl study. Hum Reprod Open 2024;2024:hoae001. 38283622 10.1093/hropen/hoae001 PMC10813743

[deag023-B58] van Duijn L , Rousian M, Laven JSE, Steegers-Theunissen RPM. Periconceptional maternal body mass index and the impact on post-implantation (sex-specific) embryonic growth and morphological development. Int J Obes (Lond) 2021;45:2369–2376. 34290384 10.1038/s41366-021-00901-7

[deag023-B59] Vellinga RE , van Bakel M, Biesbroek S, Toxopeus IB, de Valk E, Hollander A, van ‘t Veer P, Temme EHM. Evaluation of foods, drinks and diets in the Netherlands according to the degree of processing for nutritional quality, environmental impact and food costs. BMC Public Health 2022;22:877. 35501799 10.1186/s12889-022-13282-x PMC9063197

[deag023-B60] Visser M , Elstgeest LEM, Winkens LHH, Brouwer IA, Nicolaou M. Relative validity of the HELIUS Food Frequency Questionnaire for measuring dietary intake in older adult participants of the Longitudinal Aging Study Amsterdam. Nutrients 2020;12:1998. 32635636 10.3390/nu12071998 PMC7400819

[deag023-B61] White M , Arif-Pardy J, Bloise E, Connor KL. Identification of novel nutrient sensitive human yolk sac functions required for embryogenesis. Sci Rep 2024;14:29734. 39613845 10.1038/s41598-024-81061-2 PMC11607434

[deag023-B62] Wu G , Bazer FW, Cudd TA, Meininger CJ, Spencer TE. Maternal nutrition and fetal development. J Nutr 2004;134:2169–2172. 15333699 10.1093/jn/134.9.2169

[deag023-B63] Zilaitiene B , Dirzauskas M, Preiksa RT, Matulevicius V. Cigarette smoking and waiting time to pregnancy: results of a pilot study. Medicina (Kaunas) 2007;43:959–963. 18182840

[deag023-B64] Zupo R , Castellana F, Boero G, Matera E, Colacicco G, Piscitelli P, Clodoveo ML, Rondanelli M, Panza F, Lozupone M et al Processed foods and diet quality in pregnancy may affect child neurodevelopment disorders: a narrative review. Nutr Neurosci 2024;27:361–381. 37039128 10.1080/1028415X.2023.2197709

