# Supplementary material for: Periconceptional ultra-processed food consumption in women and men, fertility, and early embryonic development
Source: Hum Reprod. 2026 Mar 24;41(5):722–32. doi: 10.1093/humrep/deag023 (PMC13139660; doi:10.1093/humrep/deag023)
Supplement: deag023_Supplementary_materials_and_methods [file deag023_supplementary_materials_and_methods.pdf]

OXFORD
human reproduction

Human Reproduction, 2026, 1–1
https://doi.org/10.1093/humrep/deag023
Supplementary Data

Supplementary materials and methods

Additional information about the Cox proportional hazards models

We examined associations between maternal and paternal UPF consumption with fecundability using Cox proportional hazards models (R package *survival*). In these models, the outcome was conception, and the time variable was time to pregnancy defined as monthly cycles, with one cycle equal to 28 days. Cox models are commonly used in fecundability research because they allow estimation of probability of conception per month, while accounting for time-to-event data structures. In our analyses, all women eventually conceived during follow-up. Model assumptions were systematically evaluated. The proportional hazards assumption was assessed by using the Schoenfeld residuals, nonlinearity was assessed by Martingale residuals, and influential observations were examined by the deviance residuals. Proportional hazards violations were detected for parity, women's ethnicity, and women's BMI (combined confounder models only) and addressed by the inclusion of time-transformed covariates. The resulting hazard ratios (HRs) are interpreted as fecundability ratios (FRs), representing the relative probability of conception in each month of attempt.
