# Supplementary figures and images for: Periconceptional ultra-processed food consumption in women and men, fertility, and early embryonic development
Source: Hum Reprod. 2026 Mar 24;41(5):722–32. doi: 10.1093/humrep/deag023 (PMC13139660; doi:10.1093/humrep/deag023)

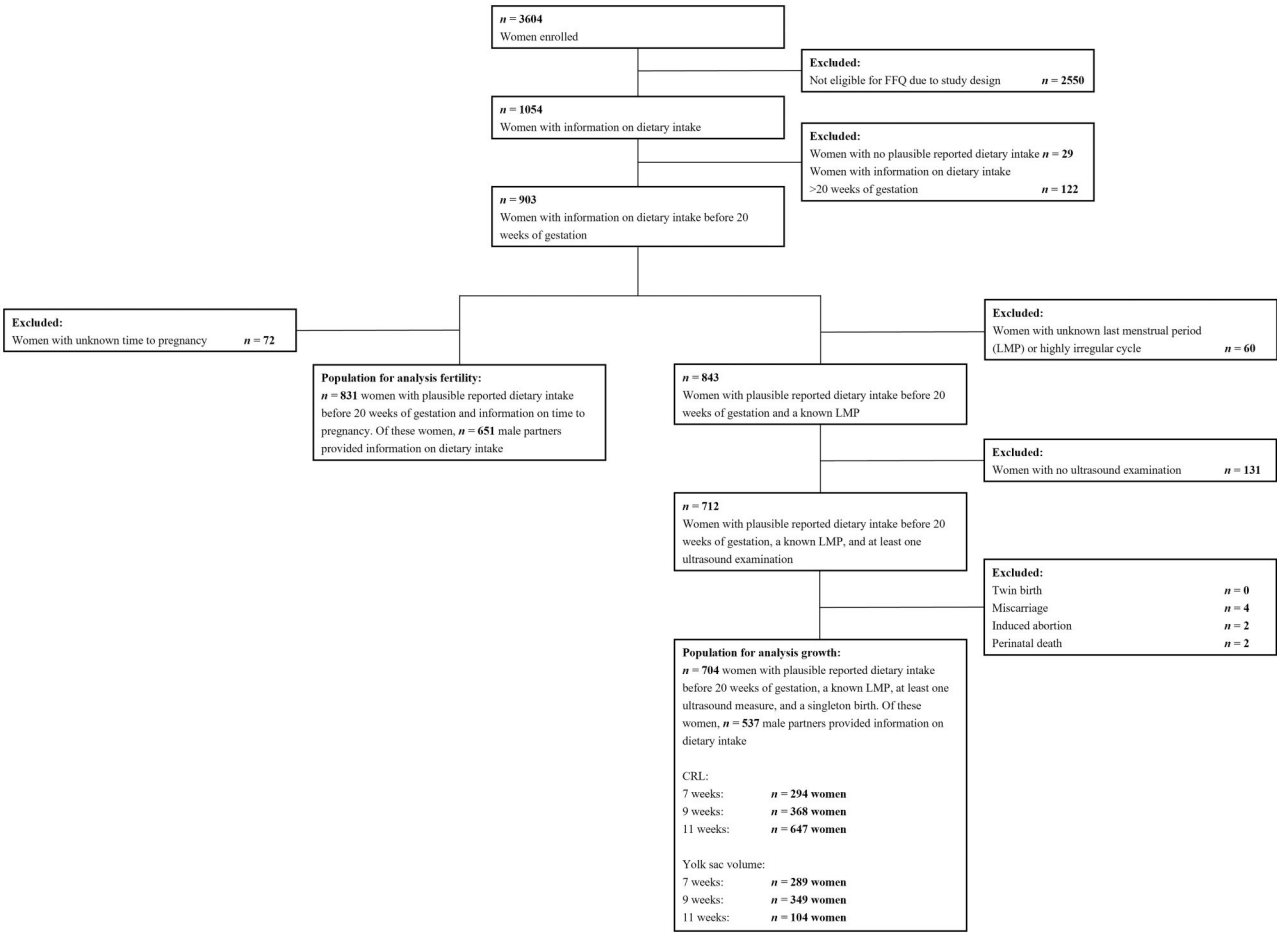

Supplementary Figure S1. Flowchart of participants included in the study.

Supplement: deag023_Supplementary_Figure_S1 [file deag023_supplementary_figure_s1.pdf]
