# Supplementary material for: Periconceptional ultra-processed food consumption in women and men, fertility, and early embryonic development
Source: Hum Reprod. 2026 Mar 24;41(5):722–32. doi: 10.1093/humrep/deag023 (PMC13139660; doi:10.1093/humrep/deag023)
Supplement: deag023_Supplementary_Figure_S2 [file deag023_supplementary_figure_s2.pdf]

OXFORD
human reproduction
Human Reproduction, 2026, 1–1
https://doi.org/10.1093/humrep/deag023
Supplementary Data

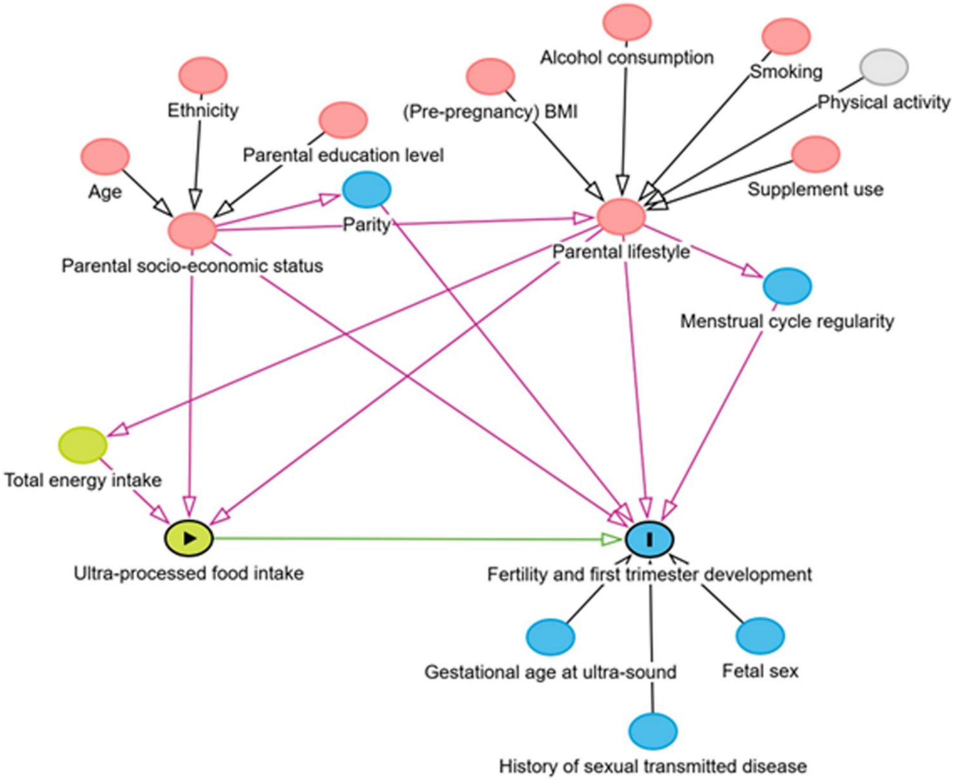

Supplementary Figure S2. Directed acyclic graph (DAG) representing the pathways between dietary ultra-processed food (UPF) consumption, fertility, and first trimester development. The DAG represents the assumed causal structure in our study on parental UPF intake, fertility, and first trimester development. Green node indicates our exposure (UPF intake) and blue node our outcomes (fertility and first trimester embryonic and yolk sac development). All other green and blue nodes are variables descendent to our exposure and outcome, respectively. Pink nodes indicate potential confounders, grey nodes unmeasured variables. Green lines indicate potential causal pathways and pink lines biased pathways.
