# Supplementary material for: Periconceptional ultra-processed food consumption in women and men, fertility, and early embryonic development
Source: Hum Reprod. 2026 Mar 24;41(5):722–32. doi: 10.1093/humrep/deag023 (PMC13139660; doi:10.1093/humrep/deag023)
Supplement: deag023_Supplementary_Table_S1 [file deag023_supplementary_table_s1.pdf]

OXFORD
human reproduction

Human Reproduction, 2026, 1–1
https://doi.org/10.1093/humrep/deag023
Supplementary Data

Supplementary Table S1. Population characteristics of the embryonic growth study population.

	Women n = 704	Men n = 537
Population characteristics		
Age at dietary assessment (years), mean (SD)	32.1 (3.9)	34.2 (5.0)
Gestational age at dietary intake assessment (weeks$^{+\text{ days}}$), median (95% range)	12^{+3} (10^{+6}, 17^{+5})	12^{+4} (10^{+6}, 20^{+0})
Ethnicity (n, %)		
Dutch	486 (69.0)	398 (74.1)
Other European	62 (8.8)	42 (7.8)
Non-European	148 (21.0)	94 (17.5)
Educational level, high (n, %)	566 (80.4)	396 (73.7)
(Pre-pregnancy) body mass index (kg/m^2), median (IQR)	23.1 (21.2, 25.4)	24.7 (22.9, 26.9)
Overweight/obesity (n, %)	197 (30.0)	242 (45.1)
Smoking during pregnancy (n, %)	75 (10.7)	69 (12.8)
Alcohol use during (before pregnancy in men) pregnancy (n, %)	114 (16.2)	487 (90.7)
Drug use during pregnancy (n, %)	15 (2.1)	72 (13.4)
Folic acid supplement use (n, %)	677 (96.2)	*na*
Parity, nulliparous (n, %)	492 (69.9)	*na*
Nausea and vomiting during early pregnancy (n, %)		
Any nausea	592 (84.1)	*na*
Any vomiting	186 (26.4)	*na*
Daily nausea and vomiting	14 (2.0)	*na*
Previously treated for a sexual transmitted disease (n, %)	134 (19.0)	*na*
Pregnancy result of infertility treatment (n, %)	81 (11.5)	*na*
Menstrual cycle regularity (n, %)		
Regular cycle (21–35 days), average usual cycle known	662 (94.0)	*na*
Regular cycle (21–35 days), average usual cycle unknown	42 (6.0)	*na*
Fetal sex, boy (n, %)	346 (49.1)	*na*
Total energy intake (kcal/day), mean (SD)	1883.6 (513.9)	2345.0 (587.7)
Carbohydrate (grams/day), mean (SD)	216.3 (64.0)	249.8 (70.3)
Protein (grams/day), mean (SD)	75.1 (22.2)	95.9 (26.5)
Fat (grams/day), mean (SD)	69.0 (22.7)	87.5 (27.6)
Fiber (grams/day), mean (SD)	22.8 (6.8)	25.9 (8.3)
UPFs (grams/day), median (IQR)	574 (410, 821)	649 (485, 899)
% UPFs (of total grams consumed), median (IQR)	22.5 (15.8, 31.3)	25.5 (18.5, 32.9)
Outcome characteristics		
**7-week ultrasound examination**		
Gestational age (weeks$^{+\text{ days}}$), median (95% range)	7^{+6} (6^{+6}, 8^{+4})	*na*
Crown-rump length (mm), mean (SD)	14.0 (4.2)	*na*
Yolk sac volume (mm^3), median (IQR)	127.2 (103.5, 150.7)	*na*
**9-week ultrasound examination**		
Gestational age (weeks$^{+\text{ days}}$), median (95% range)	9^{+5} (8^{+5}, 10^{+4})	*na*
Crown-rump length (mm), mean (SD)	29.1 (6.5)	*na*
Yolk sac volume (mm^3), median (IQR)	178.6 (148.7, 220.3)	*na*
**11-week ultrasound examination**		
Gestational age (weeks$^{+\text{ days}}$), median (95% range)	12^{+2} (10^{+6}, 13^{+3})	*na*
Crown-rump length (mm), mean (SD)	59.9 (9.2)	*na*
Yolk sac volume (mm^3), median (IQR)	208.1 (139.3, 293.1)	*na*

IQR, interquartile range; na, not applicable; UPF, ultra-processed food.
