# Supplementary material for: Periconceptional ultra-processed food consumption in women and men, fertility, and early embryonic development
Source: Hum Reprod. 2026 Mar 24;41(5):722–32. doi: 10.1093/humrep/deag023 (PMC13139660; doi:10.1093/humrep/deag023)
Supplement: deag023_Supplementary_Table_S2 [file deag023_supplementary_table_s2.pdf]

OXFORD
human reproduction

Human Reproduction, 2026, 1–1
https://doi.org/10.1093/humrep/deag023
Supplementary Data

Supplementary Table S2. Population characteristics of women according to periconceptional ultra-processed food (UPF) consumption quartiles.

	Total group n = 831	UPF quartile 1 n = 208	UPF quartile 2 n = 208	UPF quartile 3 n = 207	UPF quartile 4 n = 208	P-value
Population characteristics						
Age at enrollment (years), mean (SD)	32.1 (3.9)	32.7 (3.6)	32.9 (3.7)	31.6 (3.7)	31.4 (4.4)	<0.001
Gestational age at dietary intake (weeks^{+days}), median (95% range)	12^{+3} (10^{+6}, 18^{+3})	12^{+2} (11^{+5}, 13^{+4})	12^{+3} (11^{+6}, 14^{+4})	12^{+3}(11^{+6}, 13^{+2})	12^{+3} (11^{+6}, 13^{+5})	0.77
Ethnicity (n, %)						0.002
Dutch	570 (68.6)	133 (63.9)	143 (68.8)	152 (73.4)	142 (68.3)	
Other European	79 (9.5)	31 (14.9)	26 (12.5)	12 (5.8)	10 (4.8)	
Non-European	178 (21.4)	42 (20.2)	38 (18.3)	43 (20.8)	55 (26.4)	
Educational level, high (n, %)	675 (81.2)	187 (89.9)	176 (84.6)	170 (82.1)	142 (68.3)	<0.001
(Pre-pregnancy) body mass index (kg/m^2), median (IQR)	23.0 (21.1, 25.3)	22.7 (20.7, 24.7)	22.9 (21.1, 24.9)	23.1 (20.9, 25.8)	23.6 (21.7, 26.1)	0.004
Overweight/obesity (n, %)	222 (26.7)	43 (20.7)	49 (23.6)	61 (29.5)	69 (33.2)	0.02
Smoking before pregnancy (n, %)	315 (37.9)	70 (33.7)	85 (40.9)	71 (34.3)	89 (42.8)	0.31
Alcohol use before pregnancy (n, %)	702 (84.5)	180 (86.5)	178 (85.6)	181 (87.4)	163 (78.4)	0.046
Drug use before pregnancy (n, %)	72 (8.7)	19 (9.1)	19 (9.1)	15 (7.2)	19 (9.1)	0.87
Periconceptional folic acid supplementation (n, %)	805 (96.9)	201 (96.7)	201 (96.6)	201 (97.1)	202 (97.1)	0.91
Parity, nulliparous (n, %)	595 (71.6)	154 (74.0)	145 (69.7)	156 (75.4)	140 (67.3)	0.13
Daily nausea and vomiting during early pregnancy (n, %)	20 (2.4)	2 (1.0)	4 (1.0)	4 (1.9)	10 (4.8)	0.09
Previous miscarriage (n, %)	142 (17.1)	33 (15.9)	39 (18.3)	30 (14.5)	40 (19.2)	0.67
Previously treated for a sexually transmitted disease (n, %)	170 (20.5)	34 (16.3)	47 (20.2)	36 (17.4)	53 (25.5)	0.12
Total energy intake (kcal/day), mean (SD)	1871.5 (501.0)	1619.2 (391.9)	1870.1 (466.7)	1953.2 (483.8)	2043.9 (549.0)	<0.001
Carbohydrate (grams/day), mean (SD)	215.4 (62.1)	178.1 (45.0)	211.7 (51.3)	224.5 (58.7)	247.3 (69.6)	<0.001
Protein (grams/day), mean (SD)	74.5 (22.2)	68.9 (20.3)	75.2 (21.7)	76.8 (20.0)	77.3 (25.5)	<0.001
Fat (grams/day), mean (SD)	68.3 (22.5)	59.8 (18.4)	69.1 (23.1)	71.9 (22.2)	72.3 (24.0)	<0.001
Fiber (grams/day), mean (SD)	23 (6.9)	22.4 (6.0)	24.0 (6.5)	23.8 (7.6)	21.8 (7.1)	0.003
UPFs (grams/day), median (IQR)	563 (408, 816)	334 (274, 392)	504 (424, 583)	698 (558, 802)	1009 (815, 1222)	<0.001
% UPFs (of total grams consumed), median (IQR)	22.0 (15.7, 30.7)	12.0 (9.8, 14.0)	18.5 (17.0, 20.3)	26.0 (23.8, 28.1)	38.7 (34.1, 45.8)	<0.001
Outcome characteristics						
Time to pregnancy (months), median (IQR)	4.8 (1.2, 16.4)	4.0 (0.7, 12.5)	5.3 (1.1, 18.8)	4.8 (1.7, 20.8)	4.7 (1.1, 15.4)	0.12
Time to pregnancy ≥12 months or use of assisted reproductive technology (subfertility), (n, %)	254 (30.6)	50 (24.0)	73 (35.1)	67 (32.4)	64 (30.8)	0.09
Pregnancy as a result of fertility treatment (n, %)	108 (13.0)	23 (11.1)	32 (14.9)	29 (14.0)	24 (11.5)	0.34

P-values calculated using one-way ANOVA tests, Kruskal–Wallis tests, chi-square tests, or Fisher's exact tests.
IQR, interquartile range.
