# Supplementary material for: Periconceptional ultra-processed food consumption in women and men, fertility, and early embryonic development
Source: Hum Reprod. 2026 Mar 24;41(5):722–32. doi: 10.1093/humrep/deag023 (PMC13139660; doi:10.1093/humrep/deag023)
Supplement: deag023_Supplementary_Table_S3 [file deag023_supplementary_table_s3.pdf]

OXFORD
human reproduction
Human Reproduction, 2026, 1–1
https://doi.org/10.1093/humrep/deag023
Supplementary Data

Supplementary Table S3. Population characteristics of men according to periconceptional ultra-processed food (UPF) consumption quartiles.

	Total group n = 651	UPF quartile 1 n = 163	UPF quartile 2 n = 163	UPF quartile 3 n = 162	UPF quartile 4 n = 163	P-value
Population characteristics						
Age at enrollment (years), mean (SD)	34.2 (5.0)	34.5 (5.3)	33.8 (4.0)	34.3 (5.2)	34.1 (5.5)	0.91
Ethnicity (n, %)						0.26
Dutch	486 (74.7)	117 (71.8)	122 (69.3)	127 (77.9)	120 (73.6)	
Other European	51 (7.8)	15 (9.2)	12 (10.5)	16 (9.8)	8 (4.9)	
Non-European	111 (17.1)	29 (17.8)	29 (19.6)	19 (11.7)	34 (20.9)	
Educational level, high (n, %)	487 (74.8)	140 (85.9)	130 (79.8)	127 (78.4)	90 (55.2)	<0.001
Body mass index (kg/m^2), median (IQR)	24.6 (22.7, 26.8)	24.4 (22.7, 26.1)	24.7 (23.0, 26.7)	24.2 (22.2, 26.1)	25.6 (23.4, 27.8)	
Overweight/obesity (n, %)	286 (43.9)	62 (38.0)	73 (44.8)	64 (39.5)	87 (53.4)	<0.001
Smoking before pregnancy (n, %)	313 (48.0)	74 (45.4)	75 (46.0)	75 (46.3)	89 (54.6)	0.34
Alcohol use before pregnancy (n, %)	596 (91.6)	150 (95.1)	149 (91.4)	153 (94.4)	144 (88.3)	0.17
Drug use before pregnancy (n, %)	99 (15.2)	22 (14.1)	22 (13.5)	32 (19.8)	23 (14.1)	0.34
Total energy intake (kcal/day), mean (SD)	2349.4 (593.1)	2117.9 (531.3)	2347.1 (550.7)	2509.0 (587.0)	2424.4 (631.4)	<0.001
Carbohydrate (grams/day), mean (SD)	250.2 (69.1)	218.1 (57.9)	244.9 (56.4)	267.2 (71.2)	270.6 (76.3)	<0.001
Protein (grams/day), mean (SD)	95.7 (26.5)	91.4 (28.1)	95.6 (24.8)	99.9 (26.4)	95.8 (26.0)	0.01
Fat (grams/day), mean (SD)	88.0 (28.2)	78.2 (24.5)	88.8 (28.4)	95.2 (27.7)	89.7 (29.4)	<0.001
Fiber (grams/day), mean (SD)	26.1 (8.3)	25.9 (7.8)	27.1 (7.5)	27.6 (8.7)	23.7 (8.6)	<0.001
UPFs (grams/day), median (IQR)	643 (485, 866)	412 (334, 511)	597 (487, 675)	717 (607, 870)	1041 (815, 1294)	<0.001
% UPFs (of total grams consumed), median (IQR)	25.1 (18.4, 33.0)	15.3 (12.7, 16.8)	21.5 (20.3, 23.11)	28.2 (26.4, 30.2)	41.9 (37.3, 49.2)	<0.001

P-values calculated using one-way ANOVA tests, Kruskal–Wallis tests, chi-square tests, or Fisher's exact tests.
IQR, interquartile range.
