# Supplementary material for: Periconceptional ultra-processed food consumption in women and men, fertility, and early embryonic development
Source: Hum Reprod. 2026 Mar 24;41(5):722–32. doi: 10.1093/humrep/deag023 (PMC13139660; doi:10.1093/humrep/deag023)
Supplement: deag023_Supplementary_Table_S4 [file deag023_supplementary_table_s4.pdf]

OXFORD
human reproduction
Human Reproduction, 2026, 1–1
https://doi.org/10.1093/humrep/deag023
Supplementary Data

Supplementary Table S4. Non-response analysis comparing population characteristics of women and men with and without dietary intake data available.

	Women without dietary intake data n = 431	Women with dietary intake data n = 1054	P-value	Men without dietary intake data n = 595	Men with dietary intake data n = 902	P-value
Ethnicity (n, %)			<0.001			<0.001
Dutch	155 (44.0)	696 (67.1)		209 (44.0)	647 (72.8)	
European	45 (12.8)	101 (9.7)		52 (10.9)	71 (8.0)	
Non-European	152 (43.2)	241 (23.2)		214 (45.1)	171 (19.2)	
Educational level, high (n, %)	183 (52.4)	819 (79.4)	<0.001	225 (47.5)	644 (72.2)	<0.001
Parity, nulliparous (n, %)	152 (54.7)	719 (69.9)	<0.001	*na*	*na*	
(Pre-pregnancy) body mass index (kg/m^2), median (IQR)	24.3 (21.7, 28.3)	23.1 (21.1, 25.6)	<0.001	25.5 (23.2, 28.6)	24.7 (22.8, 26.9)	<0.001
Overweight/obesity (n, %)	114 (43.5)	290 (29.2)	<0.001	210 (58.2)	408 (45.7)	<0.001
Periconceptional folic acid supplement use (n, %)	231 (98.3)	993 (99.5)	0.07	*na*	*na*	
Smoking before pregnancy (n, %)	131 (52.6)	403 (44.0)	0.02	198 (52.9)	421 (47.9)	0.11
Alcohol before pregnancy (n, %)	189 (70.8)	845 (82.4)	<0.001	315 (82.7)	797 (90.8)	<0.001
Drug use before pregnancy (n, %)	26 (8.2)	102 (9.8)	0.4	*na*	*na*	

P-values are calculated using *t*-tests, Mann–Whitney *U*-tests, chi-square tests, or Fisher's exact tests.
IQR, interquartile range; na, not applicable.
