# Supplementary material for: Periconceptional ultra-processed food consumption in women and men, fertility, and early embryonic development
Source: Hum Reprod. 2026 Mar 24;41(5):722–32. doi: 10.1093/humrep/deag023 (PMC13139660; doi:10.1093/humrep/deag023)
Supplement: deag023_Supplementary_Table_S5 [file deag023_supplementary_table_s5.pdf]

OXFORD
human reproduction

Human Reproduction, 2026, 1–1
https://doi.org/10.1093/humrep/deag023
Supplementary Data

Supplementary Table S5. Associations of couples' ultra-processed food (UPF) consumption with fecundability and subfertility risk.

Couples' UPF consumption		Fecundability[a] Fecundability ratio (95% CI)	Subfertility[b] Odds ratio (95% CI)
Both partners low UPF consumption	n = 200	*Reference*	*Reference*
Discordant UPF consumption	n = 371	1.04 (0.86, 1.25)	1.00 (0.55, 1.83)
Both partners high UPF consumption	n = 80	1.05 (0.80, 1.39)	1.14 (0.74, 1.75)

[a]Values represent the fecundability among couples in which partners exhibit discordant UPF consumption or high UPF consumption, compared to couples with low UPF consumption. Fecundability represents the probability of conceiving within 1 month (28 days). Models were analyzed using Cox proportional hazards models. Fecundability ratios were derived from the hazard ratios of the Cox proportional hazards models.
[b]Values represent the odds of subfertility (≥12 months to conceive or use of assisted reproductive technology) among couples in which partners exhibit discordant UPF consumption or high UPF consumption, compared to couples with low UPF consumption. Models were analyzed using logistic regression models.
A threshold of <30% of total daily UPF consumption was defined as low consumption and ≥30% was classified as high consumption.
Models were adjusted for maternal and paternal age, ethnicity, educational level, alcohol use, smoking, (pre-pregnancy) body mass index, parity, and total energy intake.
