# Supplementary material for: Periconceptional ultra-processed food consumption in women and men, fertility, and early embryonic development
Source: Hum Reprod. 2026 Mar 24;41(5):722–32. doi: 10.1093/humrep/deag023 (PMC13139660; doi:10.1093/humrep/deag023)
Supplement: deag023_Supplementary_Table_S6 [file deag023_supplementary_table_s6.pdf]

OXFORD
human reproduction

Human Reproduction, 2026, 1–1
https://doi.org/10.1093/humrep/deag023
Supplementary Data

Supplementary Table S6. Associations of maternal ultra-processed food (UPF) consumption in quartiles with embryonic growth trajectories.

	Intercept CRL (SDS)	Slope CRL (SDS)
Percentage UPFs		
Quartile 1	*Reference*	*Reference*
Quartile 2	0.07 (−0.40, 0.53)	0.00 (−0.04, 0.04)
Quartile 3	−0.16 (−0.63, 0.30)	0.01 (−0.03, 0.06)
Quartile 4	−0.35 (−0.82, 0.12)	0.02 (−0.02, 0.06)

Values are regression coefficients obtained from linear mixed effects models, and reflect the gestational age-independent differences (intercepts) and the gestational age-dependent differences (slopes: change in growth per gestational visit per quartile of the dietary share of UPFs, compared to the lowest quartile).
Total number of women: 704, total number of observations: 1309.
CRL: crown-rump length; SDS, standard deviation score.
